# Constructing perfect phylogenies and proper triangulations for three-state characters

**DOI:** 10.1186/1748-7188-7-26

**Published:** 2012-09-24

**Authors:** Rob Gysel, Fumei Lam, Dan Gusfield

**Affiliations:** 1 Department of Computer Science, University of California, Davis, 1 Shields Avenue, Davis CA 95616, USA

**Keywords:** Perfect phylogeny, Chordal graph, Minimal triangulation, Minimal separator

## Abstract

In this paper, we study the problem of constructing perfect phylogenies for three-state characters. Our work builds on two recent results. The first result states that for three-state characters, the local condition of examining all subsets of three characters is sufficient to determine the global property of admitting a perfect phylogeny. The second result applies tools from minimal triangulation theory to the partition intersection graph to determine if a perfect phylogeny exists. Despite the wealth of combinatorial tools and algorithms stemming from the chordal graph and minimal triangulation literature, it is unclear how to use such approaches to efficiently construct a perfect phylogeny for three-state characters when the data admits one. We utilize structural properties of both the partition intersection graph and the original data in order to achieve a competitive time bound.

## Background

In this paper, we study the problem of constructing phylogenies, or evolutionary trees, to describe ancestral relationships between a set of observed taxa. Each taxon is represented by a sequence and the evolutionary tree provides an explanation of branching patterns of mutation events transforming one sequence into another.

We will focus on the widely studied *infinite sites* model from population genetics, in which the mutation of any character can occur at most once in the phylogeny. Without recombination, the phylogeny is a tree called a perfect phylogeny. The problem of determining if a set of binary sequences fits the infinite sites model without recombination corresponds to determining if the data can be derived on a perfect phylogeny. A generalization of the infinite sites model is the *infinite alleles* model, in which any character can mutate multiple times but each mutation of the character must lead to a distinct allele (state). Again, without recombination, the phylogeny is tree, called a *multi-state* perfect phylogeny. Correspondingly, the problem of determining if multi-state data fits the infinite-alleles model without recombination corresponds to determining if the data can be derived on a multi-state perfect phylogeny.

Dress and Steel [1] and Kannan and Warnow [2] both give algorithms that construct perfect phylogenies for three-state characters when one exists. The goal of this work is to extend the results in [3] using the minimal separators of the partition intersection graph to create a three state construction algorithm that is competitive with Dress and Steel’s algorithm.

## Notation and prior results

The input to our problem is a set of *n taxa* defined over a set of *mcharacters*$C=\{\chi^{1},\chi^{2},\ldots ,\chi^{m}\}$. We denote the states of character *χ*^*i*^ by $\left(\chi_{j}^{i}\right)$ for 0 ≤ *j* ≤ *r* − 1. A *species* is any sequence *s* = *s*_1_, *s*_2_, …, *s*_*m *_with $\left(s_{i}\in \{\chi_{0}^{i},\chi_{1}^{i},\ldots ,\chi_{r-1}^{i}\}\cup \{*\}\right)$ for *i* = 1, 2, …, *m*. The ∗ denotes a *missing value*. *χ*^*i*^ can also be considered as a function mapping species to character states, writing *χ*^*i*^(*s*) = *s*_*i*_. In this paper, every taxon is a species without missing values ( C  is also called a set of *full characters* in the literature). We will consider the set of taxa as an *n* × *m* matrix *M*, where each row corresponds to a taxon and each column corresponds to a character (or site).

The *perfect phylogeny problem* is to determine whether the taxa defined by a matrix *M* can be displayed on a tree *T* such that 

1. each taxon of *M* labels exactly one node in *T*,

2. each leaf in *T* is labeled by a taxon of *M*,

3. each node of *T* is labeled by a species,

4. for every character *χ*^*i*^ and for every state $\left(\chi_{j}^{i}\right)$ of character *χ*^*i*^, the set of all nodes in *T* labeled by species whose state of character *χ*^*i*^ is $\left(\chi_{j}^{i}\right)$ forms a connected subtree of *T*.

Any tree satisfying conditions 1 - 4 is called a perfect phylogeny for *M*. Any character satisfying condition 4 is said to be *compatible* with *T*. The general perfect phylogeny problem (with no constraints on *r*, *n*, and *m*) is NP-complete [4,5]. However, the perfect phylogeny problem becomes polynomially solvable (in *n* and *m*) when *r* is fixed. For *r* = 2, this follows from the Splits Equivalence Theorem [6,7]. For *r* = 3, Dress and Steel gave an *O*(*nm*^2^) algorithm [1] and for *r* = 3 or 4, Kannan and Warnow gave an *O*(*n*^2^*m*) algorithm [2]. For any fixed *r*, Agarwala and Fernández-Baca gave an *O*(2^3*r*^(*nm*^3^ + *m*^4^)) algorithm [8], which was improved to *O*(2^2*r*^*nm*^2^) by Kannan and Warnow [9].

### Definition 2.1

[7,10]*For a set of input taxa M, the partition intersection graph G(M) is obtained by associating a vertex for each character state and an edge between two vertices*$\left(\chi_{j}^{i}\right)$*and*$\left(\chi_{l}^{k}\right)$*if there exists a taxon s with*$\left(\chi^{i}(s)=\chi_{j}^{i}\right)$*and*$\left(\chi^{k}(s)=\chi_{l}^{k}\right)$.

Note that by definition, there are no edges in the partition intersection graph between states of the same character. It will be useful to consider the partition intersection graph *G*(*χ*^*i*^, *χ*^*j*^, *χ*^*k*^) of the submatrix of *M* defined by the three characters *χ*^*i*^, *χ*^*j*^, *χ*^*k*^.

### Definition 2.2

*A graph H is* chordal, *or* triangulated, *if there are no induced chordless cycles of length four or greater in H.*

See [11] and [12] for further details on chordal graphs.

Consider coloring the vertices of the partition intersection graph *G*(*M*) by colors 1, 2, …, *m* as follows. For each character *χ*^*i*^, assign color *i* to the vertices $\left(\chi_{0}^{i},\chi_{1}^{i},\ldots ,\chi_{r-1}^{i}\right)$. A pair of distinct vertices *u,v* of *G*(*M*) with the same color is called a *monochromatic pair*. A *proper triangulation* of the partition intersection graph *G*(*M*) is a chordal supergraph of *G*(*M*) such that every edge has endpoints with different colors. In [10], Buneman established the following fundamental connection between the perfect phylogeny problem and triangulations of the corresponding partition intersection graph.

### Theorem 2.3

[7,10]*A set of taxa M admits a perfect phylogeny if and only if the corresponding partition intersection graph G(M) has a proper triangulation.*

A triangulation of a graph *G* is *minimal* if it does not have a proper subgraph that is also a triangulation of *G*. Theorem 2.3 can be restated in terms of proper minimal triangulations of *G*(*M*) because removing edges from a proper triangulation will preserve the coloring of the graph. If *G*(*M*) has a proper triangulation *H*, then a perfect phylogeny for *M* can be constructed from a *clique tree* of *H*. T  is a clique tree for a graph *G* if 

1. the nodes of T  are in bijection with the maximal cliques of *G*,

2. for each vertex *v* of *G*, the maximal cliques containing *v* form a connected subtree of T .

That is, given a clique tree T  for a proper triangulation *H* of *G*(*M*), we label each node by its corresponding maximal clique. Because *H* is properly colored, this maximal clique includes at most one state per character and therefore defines a species. Each taxon *t* defines a clique *K*_*t*_ of size *m* in *G*(*M*), and because *H* is a triangulation of *G*(*M*), *K*_*t*_ is a clique in *H* as well. Furthermore, *H* is a proper triangulation, so *K*_*t*_ is a maximal clique of *H*. For a clique tree T , we label the node corresponding to *K*_*t*_ by *t* to obtain a perfect phylogeny for *M*. Conversely, if *M* has a perfect phylogeny *T*, then the species in *T* define a set of additional edges to obtain a proper triangulation for *G*(*M*). This is due to the following characterization of chordal graphs by the intersections of subtrees of a tree.

### Theorem 2.4

[10,13]*G is a chordal graph if and only if there is a tree T such that each vertex u of G induces a subtree T_*u*_ of T and uv is an edge of G if and only if subtrees T_*u*_ and T_*v*_ share at least one node.*

In particular, if a pair of character states appear in the same species of a perfect phylogeny for *M* but not in any input taxon of *M*, this pair defines a fill edge to add to obtain a proper triangulation of the partition intersection graph. This fill edge preserves the proper coloring because intersecting subtrees from the same character would contradict conditions 3 and 4 of the perfect phylogeny definition.

**Figure 1:**
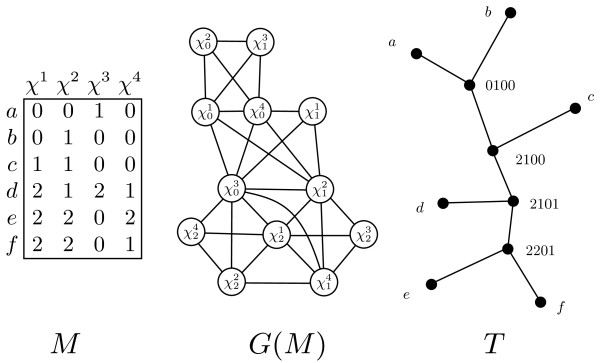
**Partition intersection graphs and perfect phylogenies.** A 3-state matrix *M*, partition intersection graph *G*(*M*), and perfect phylogeny *T*. There are no species with missing values in *T*.

To illustrate some of these notions, consider the example in Figure 1. The species with sequence 2100 defines a fill edge $\left(\chi_{2}^{1}\chi_{0}^{4}\right)$ which is not an edge of *G*(*M*) (this is the only such fill edge). Nevertheless *G*(*M*) itself is chordal, and adding this fill edge would result in a proper triangulation that is not minimal.

In recent work, it is shown that there is a complete description of minimal obstruction sets for three-state characters analogous to a well-known result on obstruction sets for binary characters (the four gamete condition) [3]. These results allow us to expand upon recent work of Gusfield [14] which uses properties of triangulations and minimal separators of partition intersection graphs to solve several problems related to multi-state perfect phylogenies.

An *(a,b)-separator* of a graph *G* is a set of vertices whose removal from *G* separates *a* and *b*. A *minimal (a,b)-separator* is an (*a,b*)-separator such that no proper subset is an (*a,b*)-separator, and a *minimal separator* is a separator that is a minimal (*a,b*)-separator for some pair of vertices *a* and *b*. For a set of vertices *X*, let *G-X* be the induced subgraph of *G* after removing vertices *X*. If *S* and *S*^*′*^ are two minimal separators of *G*, we say *S* is *parallel* to *S*^*′*^ if there is a single connected component *C* of *G* − *S*^*′*^ such that *S* ⊆ *C* ∪ *S*^*′*^ (otherwise *S* and *S*^*′ *^*cross*). A pair of vertices *a* and *b cross S* if *S* is an (*a,b*)-separator. The *neighborhood* of a set of vertices *X* is *N*(*X*) = {*v* ∈ *G* − *X* : (*u*, *v*) ∈ *E*(*G*) for some *u* ∈ *X*}. A component *C* of *G-S* is *full* if the neighborhood *N*(*C*) is equal to *S*. The following characterization of minimal separators is critical to our arguments.

### Lemma 2.5

[15]*Let S be a subset of vertices of graph G. Then S is a minimal separator of G if and only if G-S has two or more full components.*

In a colored graph, a *legal separator* is a separator such that no two vertices have the same color. Let *Δ*_*G*_ denote the minimal separators of graph *G*. For *S* ∈ *Δ*_*G*_, we *saturateS* by adding edges between every pair of vertices in *S* to create a clique. For *Q* ⊆ *Δ*_*G*_, *G*_*Q*_ denotes the graph obtained by saturating every *S* ∈ *Q*. The following theorem shows the connection between minimal triangulations and collections of parallel minimal separators of a graph.

### Theorem 2.6

(Minimal Triangulation Theorem [16-18]). *Suppose Q ⊆ Δ_*G*_ is a maximal set of pairwise parallel minimal separators of G. Then G_*Q*_ is a minimal triangulation of G and *$\left(\Delta_{G_{Q}}=Q\right)$. *Conversely, if H is a minimal triangulation of G, then Δ_*H*_ is a maximal pairwise parallel set of minimal separators of G*.

The following are necessary and sufficient conditions for the existence of a perfect phylogeny for data over arbitrary number of states. We refer the reader to [14] for the proofs.

### Theorem 2.7

(Theorem 2 (MSP) [14]). *For input M over r states (r ≥ 2), there is a perfect phylogeny for M if and only if there is a set Q of pairwise parallel legal minimal separators in G(M) such that every illegal minimal separator in G(M) is crossed by at least one separator in Q*.

### Theorem 2.8

(Theorem 3 (MSPN) [14]). *For input M over r states (r ≥ 2), there is a perfect phylogeny for M if and only if there is a set Q of pairwise parallel legal minimal separators in partition intersection graph G(M) such that every monochromatic pair of nodes in G(M) is separated by some separator in Q*.

For the special case of input *M* with characters over three states (*r* = 3), the partition intersection graph satisfies additional structure and the following theorems give necessary and sufficient conditions for the existence of a perfect phylogeny for *M*[3].

### Theorem 2.9

[3]*Given an input set M with at most three states per character (r ≤ 3), M admits a perfect phylogeny if and only if every subset of three characters of M admits a perfect phylogeny.*

Furthermore, there is an explicit description of all minimal obstruction sets to the existence of a perfect phylogeny.

### Theorem 2.10

[3]*For input M over 3-state characters, there exists a perfect phylogeny for M if and only if both of the following conditions hold: *

1. for every pair of columns of M, the partition intersection graph induced by the columns is acyclic and

**Figure 2:**
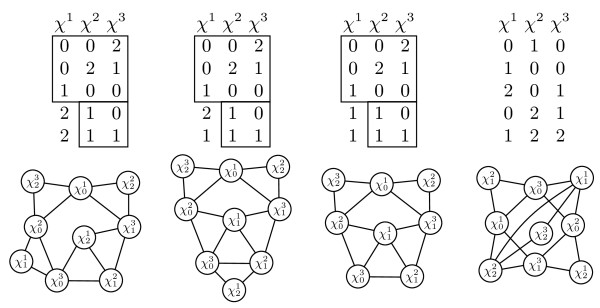
**Minimal obstruction sets.** Minimal obstruction sets for three-state characters up to relabeling. The boxes highlight the input entries that are identical for three of the obstruction sets.

*2. for every triple of columns of M, the partition intersection graphs induced by the columns does not contain any of the graphs shown in Figure *2* up to relabeling of the character states.*

This complete characterization of minimal obstruction sets allows us to simplify Theorem 2.8 in the case *r* = 3.

### Theorem 2.11

[3]*For input M on at most three states per character (r ≤ 3), there is a three-state perfect phylogeny for M if and only if the partition intersection graph for every pair of characters is acyclic and every monochromatic pair of vertices in G(M) is separated by a legal minimal separator.*

Theorem 2.11 shows that the requirement of Theorem MSPN that the legal minimal separators in *Q* be pairwise parallel can be removed for the case of input data over three-state characters. The condition in Theorem 2.11 that the input is over three state characters is necessary, as there are examples showing that the theorem does not extend to data with four-state characters.

All of the legal minimal separators for three-state input can be found in *O*(*nm*^2^) time and the algorithm to check if each monochromatic pair is separated by a legal minimal separator can be performed during the algorithm for generating the legal minimal separators (see Section “Proper triangulation algorithm”). Therefore, the 3-state perfect phylogeny *decision* problem can be solved in *O*(*nm*^2^) time using minimal separators. However, it is not clear how minimal separators can be used to solve the *construction* problem in a similar time bound. In [14], Gusfield used the minimal separator approach and integer linear programming methods to solve both the decision and construction problem for *k*-state perfect phylogeny. Since integer linear programming methods in general do not have polynomial time bounds, this naturally leads to the following question: is there an *O*(*nm*^2^) algorithm for the construction problem for 3-state perfect phylogeny using the separator approach? In this paper, we answer in the affirmative, and show that any algorithm which explicitly computes the partition intersection graph has a time bound of at least *O*(*nm* + *m*^2^).

We first study the structure of separators in the partition intersection graph for 3-state input with the goal of answering this question. We first state two lemmas from [3].

### Lemma 2.12

(Lemma 3.4 [3]). *Let M be a set of input taxa with at most three states per character, and consider any three characters χ^i^*, *χ*^*j*^, *χ*^*k*^*in M*. *If the partition intersection graph G*(*χ*^*i*^, *χ*^*j*^, *χ*^*k*^) *is properly triangulatable, then the only possible chordless cycles in G*(*χ*^*i*^, *χ*^*j*^, *χ*^*k*^) *are chordless 4-cycles, with two colors appearing once and the remaining color appearing twice.*

Lemma 2.12 implies that if a subset of three characters *χ*^*i*^, *χ*^*j*^, *χ*^*k*^ in *M* is properly triangulatable, then there is a unique set of edges *F*(*χ*^*i*^, *χ*^*j*^, *χ*^*k*^) that must be added to triangulate the chordless cycles in *G*(*χ*^*i*^, *χ*^*j*^, *χ*^*k*^). Construct a new graph *G*^*′*^(*M*) on the same vertices as *G*(*M*) with edge set $E(G(M))\cup (\underset{1\leq i<j<k\leq m}{⋃}F(\chi^{i},\chi^{j},\chi^{k}))$. *G*^*′*^(*M*) is the partition intersection graph *G*(*M*) together with additional edges to properly triangulate all chordless cycles in each *G*(*χ*^*i*^, *χ*^*j*^, *χ*^*k*^) for 1 ≤ *i* < *j* < *k* ≤ *m* (note these are the chordless 4-cycles of *G*(*M*) on three colors). In *G*^*′*^(*M*), edges from the partition intersection graph *G*(*M*) are called *E-edges* and edges that have been added as triangulation edges for some triple of columns are called *F-edges*.

### Lemma 2.13

(Lemmas 4.2, 4.3, 4.7 [3]) *Let M be a set of input taxa with at most three states per character, and suppose G*(*M*) *is properly triangulatable*. *Then G*^*′*^(*M*) *cannot contain a chordless cycle with one or more F-edges*. *If C is a chordless cycle in G*^*′*^(*M*) *with only E-edges, then C has length exactly four with four distinct colors*.

## Structure of separators

In this section, our goal is to study the relationship between minimal separators in *G*(*M*) and *G*^*′*^(*M*) when *M* is a set of taxa over 3-state characters. Our ultimate goal is to show that it suffices to consider only the legal minimal separators of *G*(*M*) while disregarding the illegal minimal separators. We first prove the following theorem on the separator structure of *G*^*′*^(*M*).

### Theorem 3.1

*Let M be a set of taxa over 3-state characters. M allows a perfect phylogeny if and only if G*^*′*^(*M*) *(the partition intersection graph G*(*M*) *together with F-edges) does not contain any illegal minimal separators.*

### Proof

Suppose *M* allows a perfect phylogeny and suppose there is an illegal minimal separator *S* in *G*^*′*^(*M*) with a monochromatic pair of vertices *u* and *v*. By Lemma 2.5, there exist two full components *C*, *D* of *G - S*, and by definition of a full component, there are paths connecting *u* and *v* in both *C*∪{*u*,*v*} and *D*∪{*u*,*v*}. Consider the shortest such paths *P*_*C*_ and *P*_*D*_ respectively (note that there are no chords within *P*_*C*_ and no chords within *P*_*D*_). Since *C* and *D* are components separated by *S*, there are no edges between *C* and *D*. Also, *u* and *v* are not adjacent in *G*^*′*^(*M*) since *u* and *v* have the same color and *G*^*′*^(*M*) contains no illegal edges. This implies the union of *P*_*C*_ and *P*_*D*_ creates a chordless cycle. By Lemma 2.13, *G*^*′*^(*M*) cannot contain any chordless cycles of length five or greater or chordless cycles with *F*-edges, so the union of the paths *P*_*C*_ and *P*_*D*_ must be a four cycle *C* and in particular, must be a cycle *u* → *x* → *v* → *x*^*′*^ → *u*, where *u* and *v* have the same color. *C* is a chordless four cycle in *G*(*M*) on at most three colors, which cannot occur since we have triangulated all such cycles by *F*-edges. This contradiction implies *S* cannot be an illegal minimal separator.

Now, suppose *G*^*′*^(*M*) does not contain any illegal minimal separators. By Theorem 2.7, graph *G*^*′*^(*M*) has a proper triangulation and since *G*(*M*) is a subgraph of *G*^*′*^(*M*), *G*(*M*) also has a proper triangulation. It follows that *M* has a perfect phylogeny. □

This suggests that analyzing the minimal separators of *G*^*′*^(*M*) suffices for 3-state construction. However, the algorithm for enumerating the minimal separators of *G*(*M*) necessary for proper triangulations in *O*(*nm*^2^) time uses *M* (rather than *G*(*M*)), and it is not clear if it is possible to extend this approach to enumerate the necessary minimal separators of *G*^*′*^(*M*). In order to use techniques in [14], the the goal of our next two results will be to describe the relationship between the minimal separators of *G*^*′*^(*M*) and the legal minimal separators of *G*(*M*) when *M* has a perfect phylogeny.

### Lemma 3.2

*Let M be a set of taxa over 3-state characters allowing a perfect phylogeny. Then H is a proper minimal triangulation of G*(*M*) *if and only if H is a minimal triangulation of G*^*′*^(*M*).

### Proof

Suppose *H* is a proper minimal triangulation of *G*(*M*). Each *F*-edge of *G*^*′*^(*M*) comes from a chordless cycle of length four on three colors (see Lemma 2.12), so this edge must appear in any proper triangulation of *G*(*M*). Hence the *F*-edges must be edges of *H*, so *G*^*′*^(*M*) ⊆ *H* and *H* is a proper triangulation of *G*^*′*^(*M*). If *H* is not minimal with respect to *G*^*′*^(*M*), there exists *H*^*′*^ such that *G*^*′*^(*M*) ⊆ *H*^*′*^ ⊂ *H* and thus *G*(*M*) ⊆ *H*^*′*^ ⊂ *H*, contradicting the minimality of *H* with respect to *G*(*M*). Thus *H* is a minimal triangulation of *G*^*′*^(*M*).

Conversely, suppose *M* allows a perfect phylogeny and *H* is a minimal triangulation of *G*^*′*^(*M*). By Theorem 2.6, *H* = *G*^*′*^(*M*)_*Q*_ for a set *Q* of maximal pairwise parallel minimal separators of *G*^*′*^(*M*), and these minimal separators must be legal by Theorem 3.1. Every edge in *H* not in *G*(*M*) is either an *F*-edge of *G*^*′*^(*M*) or a fill edge defined by *Q*, and in both cases it must be a legal fill edge. Therefore *H* is a proper triangulation of *G*(*M*). If there is some proper triangulation *H*^*′*^ of *G*(*M*) where *G*(*M*) ⊆ *H*^*′*^ ⊆ *H* then the *F*-edges of *G*^*′*^(*M*) must be edges of *H*^*′*^, otherwise *H*^*′*^ has a chordless four cycle. Thus *H*^*′*^ is a proper triangulation of *G*^*′*^(*M*), and because *H* is a proper minimal triangulation of *G*^*′*^(*M*) it must be that *H*^*′*^ = *H*. Therefore *H* is also a proper minimal triangulation of *G*(*M*). □

Let $\left(\Delta_{G(M)}^{L}\right)$ denote the set of legal minimal separators of *G*(*M*).

### Theorem 3.3

*Suppose M is a set of taxa on 3-state characters that allows a perfect phylogeny. Then the legal minimal separators of G*(*M*) *are exactly the minimal separators of G*^*′*^(*M*) *(i.e.,*$\left(\Delta_{G^{\prime }(M)}=\Delta_{G(M)}^{L}\right)$).

### Proof

Assume *M* has a perfect phylogeny. Consider a minimal separator *S* of *G*^*′*^(*M*), and suppose *Q* is a set of maximal pairwise parallel minimal separators of *G*^*′*^(*M*) with *S* ∈ *Q*. Let *H* = *G*^*′*^(*M*)_*Q*_. *H* is a minimal triangulation of *G*^*′*^(*M*) by Theorem 2.6, and *H* is a proper minimal triangulation of *G*(*M*) by Lemma 3.2. By Theorem 2.6, *Q* is precisely the set of minimal separators of *H*. Furthermore, because *H* is also a minimal triangulation of *G*(*M*), the same theorem states that *Q* is a subset of the minimal separators of *G*(*M*). Therefore *S* ∈ *Δ*_*G*(*M*)_, so $\left(\Delta_{G^{\prime }(M)}\subseteq \Delta_{G(M)}\right)$. Each minimal separator of *G*^*′*^(*M*) is legal by Theorem 3.1. Hence $\left(\Delta_{G^{\prime }(M)}\subseteq \Delta_{G(M)}^{L}\right)$.

Conversely, let $\left(S\in \Delta_{G(M)}^{L}\right)$. First we show that if no *F*-edge *f * of *G*^*′*^(*M*) crosses *S* (i.e. *f* = *xy* where *S* separates *x* and *y*), then *S* is a minimal separator of *G*^*′*^(*M*). Let *C* be a connected component of *G*(*M*) − *S*. *C* is still connected in *G*^*′*^(*M*), and because no *F*-edge of *G*^*′*^(*M*) crosses *S*, $\left(N_{G^{\prime }(M)}(C)\subseteq S\right)$. Hence *C* is a connected component of *G*^*′*^(*M*) − *S*. Further, we have only added edges to obtain *G*^*′*^(*M*), so $\left(N_{G(M)}(C)\subseteq N_{G^{\prime }(M)}(C)\right)$. Therefore if *C* is a full component of *G*(*M*) − *S* we have $\left(N_{G(M)}(C)=N_{G^{\prime }(M)}(C)=S\right)$, and it is also a full component of *G*^*′*^(*M*)−*S*. By Lemma 2.5, *S* is a minimal separator of *G*^*′*^(*M*).

Now consider a minimal separator *S*^*′*^ of *G*(*M*). If an *F*-edge *f* = *xy* crosses *S*^*′*^, there is a four cycle *x* → *u* → *y* → *v* → *x* in *G*(*M*) with monochromatic pair *u,v*, and further, *u*,*v* ∈ *S*^*′*^. Hence *S*^*′*^ is illegal, and any legal minimal separator of *G*(*M*) is not crossed by any *F*-edge. From our previous argument, this implies $\Delta_{G(M)}^{L}\subseteq \Delta_{G^{\prime }(M)}$. Therefore $\Delta_{G^{\prime }(M)}=\Delta_{G(M)}^{L}$. □

The second half of the proof of Theorem 3.3 proves the following.

### Corollary 3.4

*Suppose M is a set of taxa on 3-state characters that allows a perfect phylogeny. If*$S\in \Delta_{G^{\prime }(M)}^{L}$*then C is a connected component of G*(*M*) − *S if and only if C is a connected component of G*^*′*^(*M*) − *S*.

We now prove the main result of this section.

### Theorem 3.5

*Suppose M is a set of taxa on 3-state characters. Then M has a perfect phylogeny if and only if any maximal pairwise parallel set of legal minimal separators Q of G*(*M*) *induces a proper minimal triangulation G*(*M*)_*Q *_*of G*(*M*).

### Proof

First, suppose that *M* has a perfect phylogeny, and let *Q* be a maximal pairwise parallel set of legal minimal separators of *G*(*M*). We show that *G*(*M*)_*Q*_ is a proper triangulation of *G*(*M*). By Theorem 3.3, *Q* is a maximal set of minimal separators of *G*^*′*^(*M*), and they are pairwise parallel because the connected components of each minimal separator in *Q* are the same in *G*(*M*) and *G*^*′*^(*M*) (Corollary 3.4). Hence *H* = *G*^*′*^(*M*)_*Q*_ is a minimal triangulation of *G*^*′*^(*M*) with minimal separator set *Q* (Theorem 2.6), and by Lemma 3.2, *H* is a proper minimal triangulation of *G*(*M*). Because *Δ*_*H*_ = *Q*, Theorem 2.6 implies *Q* is a maximal pairwise parallel set of minimal separators of *G*(*M*) and therefore *H* = *G*(*M*)_*Q*_. Thus *H* = *G*(*M*)_*Q*_ is a proper minimal triangulation of *G*(*M*).

For the converse, pick any maximal pairwise parallel set of legal minimal separators *Q* of *G*(*M*) that induces a proper minimal triangulation *G*(*M*)_*Q*_ of *G*(*M*). Then *M* has a perfect phylogeny by Theorem 2.3. □

## Proper triangulation algorithm

In this section, we build on techniques developed in [14] to generate the minimal separators of *G*^*′*^(*M*) and their parallel relations in *O*(*nm*^2^) time. This will allow us to use a greedy approach to pick a maximal pairwise parallel set of legal minimal separators. These minimal separators will then define a set of fill edges for a proper minimal triangulation, and a perfect phylogeny will be constructed in the form of a clique tree using Maximum Cardinality Search (MCS).

### Lemma 4.1

[14]*Let Q be a set of maximal pairwise parallel legal minimal separators of a partition intersection graph G*(*M*). *Then for each S* ∈ *Q*, |*S*| < *m*.

Define $\Delta_{G(M)}^{*}=\{S\in \Delta_{G(M)}^{L}:|S|<m\}$. We first state our algorithm and then analyze the running time of each step.

### Algorithm: proper triangulation for 3-state characters

1. Stop if there is a pair of characters whose partition intersection graph contains a cycle.

2. Compute $\Delta_{G(M)}^{*}$ using proper clusters.

3. Stop if there is a monochromatic pair not separated by any legal minimal separator.

4. Compute the crossing relations for $\Delta_{G(M)}^{*}$.

5. Greedily construct a maximal pairwise parallel subset *Q* of $\Delta_{G(M)}^{*}$; stop if *Q* has more than 2*n* − 3 minimal separators.

6. Add edges to *G*(*M*) to make each *S* ∈ *Q* a clique. Call this graph *G*_*Q*_.

7. Use MCS to construct a clique tree for *G*_*Q*_.

We proceed with a series of lemmas that will be used in Theorem 4.11 to show that each step is *O*(*nm*^2^). The following simple observation is important for many of our time bounds.

#### Observation 4.2

*Let M be a set of taxa whose characters have at most three states. Then G*(*M*) *has O*(*m*) *vertices (one vertex per state of each character) and O*(*m*^2^) *edges.*

**Figure 3:**
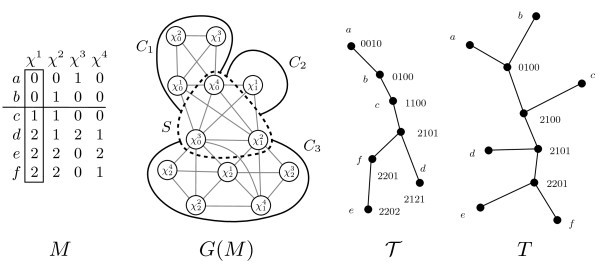
**Minimal separators and proper clusters.** In this figure, the bipartition *ab*|*cdef* gives rise to the proper clusters *ab* and *cdef*. The shared character states $\left(\chi_{1}^{2},\chi_{0}^{3},\chi_{0}^{4}\right)$ form a legal minimal separator *S* in *G*(*M*). *G*(*M*) − *S* has three connected components, of which two are full (components *C*_1_ and *C*_2_). The *S*-partition gives rise to the bipartition because *t*(*C*_1_) = {*a*, *b*} and *t*(*C*_2_) ∪ *t*(*C*_3_) = {*c*, *d*, *e*, *f*}. T  is a clique tree for *G*(*M*) (in this case, *G*(*M*) happens to be chordal). *T* is obtained from T  by resolving the nodes labeled *b, c, f*. Note that *S* is represented in T  on edge *bc* because $\left(\left\{\chi_{0}^{1},\chi_{1}^{2},\chi_{0}^{3},\chi_{0}^{4}\}\cap \{\chi_{0}^{1},\chi_{1}^{2},\chi_{0}^{3},\chi_{0}^{4}\}=\{\chi_{1}^{2},\chi_{0}^{3},\chi_{0}^{4}\right\}\right)$. For a clique tree T  of a chordal graph, every minimal separator of the chordal graph behaves this way [11,21]. In this sense, legal minimal separators are analagous to splitting vectors.

Step 2 of the algorithm uses concepts from [2,8,9,14], which we detail here for completeness. A *proper cluster* is a bipartition of the taxa (i.e. the taxa are split into two disjoint nonempty sets) such that each character shares at most one state across the bipartition, and at least one character is not shared across this bipartition [8,9]. There are *O*(*m*) proper clusters when *r* is fixed. In particular, suppose *χ* is not shared across the bipartition of a proper cluster. Then the proper cluster also creates a bipartition of *χ*’s character states (see Figure 3). Hence, we can compute the set of proper clusters by exhaustively checking, for each character, if some bipartition of its states split the taxa into a proper cluster (there are *O*(2^*r*^) ways to split each character). 

Proper clusters generate the minimal separators in $\Delta_{G(M)}^{*}$ as follows [14]. For a connected component *C* of *G*(*M*) − *S*, let *t*(*C*) be the set of taxa with character-state $\left(\chi_{j}^{i}\right)$ for at least one $\left(\chi_{j}^{i}\in C\right)$. We will refer to the set of *t*(*C*) determined by the connected components of *G*(*M*) − *S* as the *S*-partition of the taxa. Recall *S* has at most *m* − 1 vertices by Lemma 4.1, so every taxon must have a character-state that is not a vertex of *S*. Hence no taxon can have all of its character-states as vertices of *S*. Additionally, each taxon defines a clique, so it cannot have vertices in more than one connected component of *G*(*M*) − *S* (this would define an edge between connected components). By Lemma 2.5, *G*(*M*) − *S* has two or more full components *C*_1_ and *C*_2_. Place *t*(*C*_1_) and *t*(*C*_2_) in separate parts of the bipartition, then for the remaining connected components *C* of *G*(*M*) − *S* add *t*(*C*) to either part. This defines a bipartition where the shared character states (known as the splitting vector [9]) are exactly the vertices of *S*. To see this, suppose a character-state $\left(\chi_{j}^{i}\right)$ is a vertex of *S*. Because *C*_1_ is a full component, there is a vertex $\left(\chi_{j_{0}}^{i_{0}}\in C_{1}\right)$ adjacent to $\left(\chi_{j}^{i}\right)$. Because these vertices are adjacent, $\left(\chi_{j_{0}}^{i_{0}}\right)$ and $\left(\chi_{j}^{i}\right)$ appear in the same row of *M*, which in turn is a taxon *t*_1_ of *t*(*C*_1_). Similarly, there exists *t*_2_ ∈ *t*(*C*_2_) such that *χ*_*i*_(*t*_2_) = *j*, so $\left(\chi_{j}^{i}\right)$ is shared in the bipartition. See Figure 3 for an illustration of these concepts. This implies that $\left|\Delta_{G(M)}^{*}|=O(m\right)$. The following two lemmas are special cases of those found in [14].

#### Lemma 4.3

[14]*For any set of taxa M on 3-state characters*, $\Delta_{G(M)}^{*}$*can be computed in O*(*nm*^2^) *time. Further*, $\left|\Delta_{G(M)}^{*}|=O(m\right)$.

#### Proof

Our previous discussion proves that $\Delta_{G(M)}^{*}$ has at most *O*(*m*) minimal separators, so we focus on the running time. Let *g* be a proper cluster with splitting vector *x* and let *S*_*x*_ be the vertices of *G*(*M*) appearing as character-states in *x*. Define the equivalence relation *g*/*x* by the transitive closure of the relation *tRt*^*′*^ if and only if there is a character *χ*^*i*^ where *χ*^*i*^(*t*) = *χ*^*i*^(*t*^*′*^) = *j* and $\left(\chi_{j}^{i}\right)$ is not a shared character state in *x*; calculating *g*/*x* takes *O*(*nm*) time [9]. Given an equivalence class *t* of *g* / *x*, the vertices $\left(\left\{\chi_{j}^{i}\notin S_{x}|\chi^{i}(t^{\prime })=j\;\text{for some}\;\;t^{\prime }\in \;[t]\right\}\right)$ are a connected component of *G*(*M*) − *S*_*x*_, and every connected component can be described in this way. For a connected component *C* of *G*(*M*) − *S*_*x*_, the size of its neighborhood can be calculated using the *t*(*C*) rows of *M* (i.e. for *t* ∈ *t*(*C*), count the character states of *t* also in *x*, being careful not to overcount). $S_{x}\in \Delta_{G(M)}^{*}$ if and only if there are distinct equivalence classes *t* and *t*^*′*^ that share all character states in *x*. For each equivalence class, we examine each taxon once, so this requires a single pass through every row of *M* and can be done in *O*(*nm*) time per proper cluster, so step 2 takes *O*(*nm*^2^) time. □

In the proof of Lemma 4.3, we showed how to compute the *S*-partition of the taxa for $S\in \Delta_{G(M)}^{*}$ in *O*(*nm*) time. It is now easy to calculate the connected components of *G*(*M*) − *S*: if *t*(*C*) is part of the *S*-partition, then *C* is obtained by listing the character-states that appear in at least one *t* ∈ *t*(*C*) but not in *S*. This proves the following.

#### Lemma 4.4

[14]*Let M be a set of 3-state taxa and*$S\in \Delta_{G(M)}^{*}$. *There is an O*(*nm*) *algorithm that calculates the connected components of G*(*M*) − *S and determines which of these connected components is full.*

Before discussing the running time required to compute crossing relations, we first state two structural lemmas on minimal separators; the second follows from a lemma in [19].

#### Lemma 4.5

[18]*Let S and S*^*′ *^*be non-parallel minimal separators. Then for each full component C of G − S*^*′*^, *S has a vertex in C*.

#### Lemma 4.6

(Lemma 3.10, [19]). *Let S and S*^*′ *^*be two minimal separators of a graph G. Then S and S*^*′ *^*are parallel if and only if there exists a full component C*_*S *_*of G − S and a connected component*$\left(C_{S^{\prime }}\right)$*of G − S*^*′ *^*such that*$C_{S}\subseteq C_{S^{\prime }}$.

Because of the slight change from Lemma 3.10 in [19] and for completeness, we give a proof of Lemma 4.6.

#### Proof

Suppose *S* and *S*^*′*^ are parallel. Since *S* is a minimal separator, there are at least two full components in *G* − *S* and because *S*^*′*^ is parallel to *S*, there is a full component *C*_1_ of *G* − *S* that does not intersect *S*^*′*^. *C*_1_ is connected in *G* − *S*^*′*^, so there is a connected component *C* of *G* − *S*^*′*^ containing *C*_1_.

Now, suppose there are *C*_*S*_ and $\left(C_{S^{\prime }}\right)$ satisfying the conditions of the lemma. Then $S\subseteq N(C_{S})\subseteq C_{S^{\prime }}\cup N(C_{S^{\prime }})\subseteq C_{S^{\prime }}\cup S^{\prime }$, implying that *S* and *S*^*′*^are parallel. □

#### Lemma 4.7

*There is an O*(*nm*^2^) *algorithm to calculate the crossing relations of*$\Delta_{G(M)}^{*}$.

#### Proof

Let $S,\;S^{\prime }\in \Delta_{G(M)}^{*}$. We begin by showing that *S* and *S*^*′*^ are parallel if and only if there is a full component *C* of *G*(*M*) − *S* and connected component *C*^*′*^ of *G*(*M*) − *S*^*′*^ such that *t*(*C*) ⊆ *t*(*C*^*′*^) (i.e. *t*(*C*) is contained in a single part of the *S*^*′*^−partition). Suppose *S* and *S*^*′*^ are parallel. From Lemma 4.6, there are connected components *C* of *G*(*M*) − *S* and *C*^*′*^ of *G*(*M*) − *S*^*′*^ such that *C* ⊆ *C*^*′*^ and consequently *t*(*C*) ⊆ *t*(*C*^*′*^).

Conversely, assume that *S* and *S*^*′*^are not parallel. Let *C*_1_ be a full component of *G*(*M*) − *S* and *C*_2_ be a full component of *G*(*M*) − *S*^*′*^. By Lemma 4.5, there is a vertex $v\in C_{1}\cap S^{\prime }$, and because *C*_2_ is full, there is a *u* ∈ *C*_2_ ∩ *N*(*v*). The taxa form an edge clique cover for *G*(*M*), so there is a taxon *t* having both character states corresponding to *u* and *v*. Note *v* ∈ *C*_1_ so *t* ∈ *t*(*C*_1_) and *u* ∈ *C*_2_ so *t* ∈ *t*(*C*_2_). *S*^*′*^ has at least two full components, and repeating this argument yields another full component $\left(C_{2}^{\prime }\neq C_{2}\right)$ of *G*(*M*) − *S*^*′*^ such that $t(C_{1})\cap t(C_{2}^{\prime })\neq \emptyset$. Thus *t*(*C*_1_) shares at least one taxon with at least two parts of the *S*^*′*^−partition, so *t*(*C*_1_) is not contained within any single part of the *S*^*′*^−partition. This proves our characterization of parallel minimal separators of $\Delta_{G(M)}^{*}$.

It suffices to check for each full component *C* of *G*(*M*) − *S* and connected component *C*^*′*^ of *G*(*M*) − *S*^*′*^ if *t*(*C*) ⊆ *t*(*C*^*′*^). There are *O*(*m*^2^) pairs of legal minimal separators, and this check takes *O*(*n*) time (*O*(*nm*^2^) time overall) when the *S*-partition has been calculated for each $S\in \Delta_{G(M)}^{*}$. □

**Figure 4:**
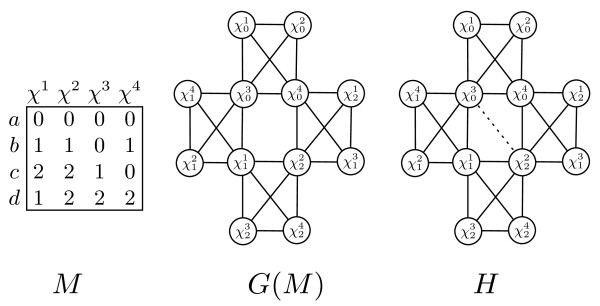
**Bounding minimal separators of proper minimal triangulations.** An example of the bound in Lemma 4.8. *H* has five minimal separators, obtained from every pair of vertices from the set $\left(\left\{\chi_{1}^{1},\chi_{2}^{2},\chi_{0}^{3},\chi_{0}^{4}\right\}\right)$ except $\left(\left\{\chi_{1}^{1},\chi_{0}^{4}\right\}\right)$. There are four taxa, so Lemma 4.8 gives an upper bound of five minimal separators. Therefore, the bound in Lemma 4.8 is tight for *n* = 4 taxa.

It is critical for our time bound that any proper minimal triangulation of *G*(*M*) have *O*(*n*) minimal separators because this impacts the computation of edges contained in the proper minimal triangulation. A result bounding the number of minimal separators in an earlier version of this paper (Lemma 7 in [20]) was incorrect, as demonstrated in Figure 4. We present a corrected bound for the number of minimal separators in the following Lemma. 

#### Lemma 4.8.

*Suppose that H is a proper minimal triangulation of G*(*M*). *Then H has at most 2n − 3 minimal separators.*

#### Proof

Let T  be a clique tree of *H*. Recall that the nodes of T  are in bijection with the maximal cliques of *H*. To make this correspondence explicit, for each node *x* of T  we will write *K*_*x*_ to mean the maximal clique of *H* that corresponds to *x*. A classic result in chordal graph theory says that if *S* ∈ *Δ*_*H*_, there is an edge *xy* of T  such that *S* = *K*_*x*_ ∩ *K*_*y*_[11,21]. Therefore the number of minimal separators in *H* is at most the number of edges of T .

First, consider any leaf *a* of T . We claim that *K*_*a*_ contains a vertex of *G* that is not in any other maximal clique of *G* (this fact is well known in the chordal graph literature [22], but we prove it here for completeness). Suppose *a*^*′*^is the neighbor of *a* in T . By maximality, $\left(K_{a}⊄K_{a^{\prime }}\right)$ so there is a vertex *v* of *H* that is contained in *K*_*a*_ but not contained in $\left(K_{a^{\prime }}\right)$. If *v* is contained in a maximal clique of *G* that is not *K*_*a*_, then the second property of clique trees implies that $\left(v\in K_{a^{\prime }}\right)$ as well. Hence *v* is only contained in *K*_*a*_, proving the claim. Further, *v* is some character-state $\left(\chi_{j}^{i}\right)$, and there is a taxon *t* of *M* such that *χ*^*i*^(*t*) = *j*. Taxon *t* can only label *a* because no other node of T  corresponds to a maximal clique that contains $\left(\chi_{j}^{i}\right)$. Thus for each leaf of T  there is a unique taxon that labels it.

To complete the proof, we show a similar result for internal nodes of T  with degree two. Let *z* be such a node with neighbors *z*_1_ and *z*_2_. If *z* contains a vertex that is only contained in *z*^*′*^s maximal clique *K*_*z*_, our previous argument shows that *z* can be labeled by a unique taxon. Suppose this is not the case. Let $S_{i}=K_{z}\cap K_{z_{i}}$ for *i* = 1, 2. It must be that *K*_*z*_ = *S*_1_ ∪ *S*_2_ because we are considering the case when *K*_*z*_ does not contain a unique vertex. Further, we cannot have *S*_1_ ⊆ *S*_2_ since otherwise $\left(K_{z}=S_{2}\subseteq K_{z_{2}}\right)$ would not be maximal. Similarly, *S*_2_ ⫅ *S*_1_. Pick *u*_1_ ∈ *S*_1_ − *S*_2_ and *u*_2_ ∈ *S*_2_ − *S*_1_, noting that $\left(u_{1}\notin K_{z_{2}}\right)$ and $\left(u_{2}\notin K_{z_{1}}\right)$. We argue that *K*_*z*_ is the only maximal clique containing both *u*_1_ and *u*_2_. This is because if any other maximal clique *K* contains both vertices, then either $\left(K_{z_{1}}\right)$ or $\left(K_{z_{2}}\right)$ is on the path from *K*_*z*_ to *K* in T  (*K* has degree two) and by the second property of clique trees, this maximal clique also contains both vertices. Further, because each *S* ∈ *Δ*_*H*_ is of the form *S* = *K*_*x*_ ∩ *K*_*y*_ for an edge *xy* of T , there is no minimal separator of *H* containing both *u*_1_ and *u*_2_. By Theorem 2.6, $\left(u_{1}u_{2}=\chi_{j_{1}}^{i_{1}}\chi_{j_{2}}^{i_{2}}\right)$ is an edge of *G*(*M*) (i.e. it is not a fill edge) because *H* is a minimal triangulation of *G*(*M*), so all fill edges come from saturating each *S* ∈ *Δ*_*H*_. Therefore there is a taxon *t*^*′*^ of *M* such that $\left(\chi^{i_{1}}(t^{\prime })=j_{1}\right)$ and $\left(\chi^{i_{2}}(t^{\prime })=j_{2}\right)$. As in the unique vertex case, *z* is the unique node with label *t*^*′*^.

Therefore any node of T  with degree at most two is labeled by a unique taxon, implying there are at most *n* such nodes. Any tree containing at most *n* leaves and internal nodes of degree two has at most 2*n* − 3 edges. Hence T  has at most 2*n* − 3 edges, and in turn *H* has at most 2*n* − 3 minimal separators, proving the bound. □

#### Remark

The proof of Lemma 4.8 requires minimality of the triangulation, but it does not require that *M* lacks missing values or that the number of states for each character is bounded.

This Lemma along with the fact that each $\left(S\in \Delta_{G(M)}^{*}\right)$ has fewer than *m* vertices gives the following result.

#### Lemma 4.9

*Suppose that H is a proper minimal triangulation of G*(*M*) *obtained by saturating a maximal pairwise parallel legal set of minimal separators Q*. *Then H has O*(*n*) *minimal separators, O*(*m*) *vertices, and O*(*m*^2^) *edges. Furthermore, H can be calculated in O*(*nm*^2^) *time.*

#### Proof

The minimal separator bound follows from Lemma 4.8, and the vertex and edges bounds follow from Observation 4.2 and the fact that *H* and *G*(*M*) have the same vertex set. In order to calculate *H*, we must calculate the fill edge set *E*(*H*) − *E*(*G*(*M*)). Recall that, by Theorem 2.6, the fill edges of *H* are obtained by saturating each minimal separator in *Q*. Each *S* ∈ *Q* has fewer than *m* vertices by Lemma 4.1 and |*Q*| = *O*(*n*) by Lemma 4.8. It is straightforward to check for each *S* ∈ *Q* and each pair *u*, *v* ∈ *S* if *uv* defines a fill edge with an amortized running time of *O*(*nm*^2^). □

In [23], Tarjan and Yannakakis developed Maximum Cardinality Search (MCS), which recognizes chordal graphs in linear time. Blair and Peyton [11] showed how MCS can be used to construct a clique tree for a chordal graph while retaining the linear time bound.

#### Lemma 4.10

[11]*Let G be a chordal graph. Then Maximum Cardinality Search (MCS) can be implemented to produce a clique tree*T *of G with running time O*(|*V*(*G*)| + |*E*(*G*)|).

Combining these lemmas show that our minimal separator algorithm for constructing perfect phylogenies for *r* = 3 is competitive with the algorithm of Dress and Steel [1], giving our main result.

#### Theorem 4.11

*The algorithm Proper Triangulation for 3-State Characters runs in O*(*nm*^2^) *time.*

#### Proof

The first step can be implemented in *O*(*m*^2^) time as follows. Each pair of characters has a partition intersection graph with at most six vertices, and it is straightforward to check for cycles. There are *O*(*m*^2^) such pairs of characters. Lemma 4.3 states that step two takes *O*(*nm*^2^) time. For the third and fourth step, we first compute the connected components of *G*(*M*) − *S* for each $S\in \Delta_{G(M)}^{*}$. Lemmas 4.3 and 4.4 tell us there are *O*(*m*) computations that require *O*(*nm*) time, so computing all the sets of connected components takes *O*(*nm*^2^) time. There are *O*(*m*) monochromatic pairs (three pairs per character), and for each monochromatic pair $\left(\chi_{i_{1}}^{i},\chi_{i_{2}}^{i}\right)$ we check the connected components of each $\left(S\in \Delta_{G(M)}^{*}\right)$ and ensure at least one of these minimal separators is a $\left(\left(\chi_{i_{1}}^{i},\chi_{i_{2}}^{i}\right)\right)$-separator. Hence step three takes *O*(*m*^2^) time. Lemma 4.7 shows that step four has a running time of *O*(*nm*^2^). Step five runs in *O*(*nm*) time due to the bounds in Lemmas 4.3 and 4.8. That is, after picking a minimal separator *S* to be in *Q*, there are *O*(*m*) minimal separators that can cross *S* and we repeat this process *O*(*n*) times to construct *Q*. Constructing *G*_*Q*_ was shown to take *O*(*nm*^2^) time in Lemma 4.9. Lemma 4.9 shows that *O*(|*V*(*G*_*Q*_)| + |*E*(*G*_*Q*_)|) = *O*(*m*^2^) so using MCS in step 7 takes *O*(*m*^2^) time. Hence each step and the pre-processing for each step takes at most *O*(*nm*^2^) time, so the algorithm takes at most *O*(*nm*^2^) time. □

## Large partition intersection graphs

Ideally, one would like to find an *O*(*n*^2^*m*) or *O*(*nm*) algorithm for 3-state perfect phylogeny (i.e., *m* is square-free). In this section, we will construct a family of 3-state matrices *M* that have a perfect phylogeny and *Λ*(*m*^2^) edges in *G*(*M*). This discourages attempts to improve our time bound using an approach that explicitly computes the partition intersection graph.

Any 3-state character compatible with a perfect phylogeny can be obtained from choosing any two edges of the phylogeny, removing them, and using the three resulting subtrees to define each taxon’s state for that character. 2-state characters are obtained in a similar manner, removing a single edge instead of two edges. Therefore, if a 3-state matrix *M* with distinct columns (up to relabeling) has a perfect phylogeny, m=O(n2)=O(n2).

**Figure 5:**
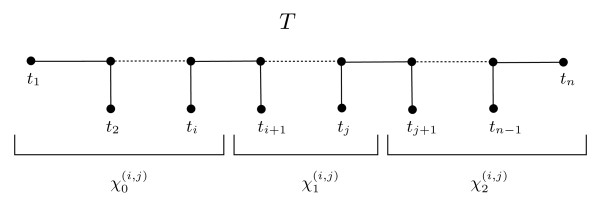
**Characters of a perfect phylogeny with a large partition intersection graph.** A 3-state character created using “intervals” of taxa from a fully resolved tree *T*. The 0^*th*^ piece of *χ*^(*i*,*j*)^ is the interval $\left(\chi_{0}^{\left(i,j\right)}\right)$.

Consider the tree *T* with taxa *t*_1_, *t*_2_, …, *t*_*n*_ as depicted in Figure 5, and suppose *i* < *j*. We construct the character *χ*^(*i*,*j*)^ using the partition {*t*_1_, *t*_2_, …, *t*_*i*_}, {*t*_*i* + 1_, *t*_*i* + 2_, …, *t*_*j*_}, {*t*_*j* + 1_, *t*_*j* + 2_, …, *t*_*n*_} as in Figure 5. Each set in the partition is called the cell 0, cell 1, and cell 2 of *χ*^(*i*,*j*)^, respectively. That is, *χ*^(*i*,*j*)^(*t*_1_) = 0, *χ*^(*i*,*j*)^(*t*_*i* + 1_) = 1, *χ*^(*i*,*j*)^(*t*_*j* + 1_) = 2, and so on. Let *M*^∗^ be the matrix whose columns are the characters *χ*^(*i*,*j*)^ for 1 ≤ *i* < *j* < *n*. *T* is clearly a perfect phylogeny for *M*^∗^, and m=n−12=Λ(n2). Next, we show that *G*(*M*^∗^) has *Λ*(*m*^2^) edges.

### Observation 5.1

*Let χ*^(*i*,*j*) ^*and*$\left(\chi^{\left(i^{\prime },j^{\prime }\right)}\right)$*be distinct characters of M*^∗^. *Then*$\left(\chi_{k}^{\left(i,j\right)}\chi_{k^{\prime }}^{\left(i^{\prime },j^{\prime }\right)}\right)$*is an edge of G*(*M*^∗^) *iff cell k of χ*^(*i*,*j*) ^*and the cell k*^*′*^*of*$\left(\chi^{\left(i^{\prime },j^{\prime }\right)}\right)$*have a non-empty intersection (i.e. share a taxon).*

For example, the cell 1 of *χ*^(3,5)^ and cell 1 *χ*^(4,6)^ share taxon *t*_5_ so $\left(\chi_{1}^{\left(3,5\right)}\chi_{1}^{\left(4,6\right)}\right)$ is an edge in *G*(*M*^∗^). In contrast, cell 0 of *χ*^(3,5)^ and cell 1 of *χ*^(4,6)^ do not share any taxa, so $\left(\chi_{0}^{\left(3,5\right)}\chi_{1}^{\left(4,6\right)}\right)$ is not an edge in *G*(*M*^∗^). Consider the characters *χ*^(*i*,*j*)^ and $\left(\chi^{\left(i^{\prime },j^{\prime }\right)}\right)$ for distinct *i*, *i*^*′*^, *j*, and *j*^*′*^. There are at least n4 pairs of these characters, and each such pair provides at least one edge to *G*(*M*^∗^) because both cell 0 of *χ*^(*i*,*j*)^ and cell 0 of $\left(\chi^{\left(i^{\prime },j^{\prime }\right)}\right)$ share *t*_1_. Therefore *G*(*M*^∗^) has at least *o*(*n*^4^) = *o*(*m*^2^) edges. There are at most m2 edges in any partition intersection graph, so *G*(*M*^∗^) has *Λ*(*m*^2^) edges, and reading each entry of *M* to compute *G*(*M*) requires at least *nm* time. Hence any construction algorithm that explicitly computes the partition intersection graph requires at least *O*(*nm* + *m*^2^) time.

## Conclusions

We have demonstrated how to use the minimal separator approach introduced in [14] to construct a perfect phylogeny for 3-state data in *O*(*nm*^2^) time. We also constructed a 3-state matrix *M* with a perfect phylogeny that has *Λ*(*m*^2^) edges. Thus, any explicit analysis of the edges of *G*(*M*) or of a proper triangulation of *G*(*M*) is inadequate to speed up our approach. Faster proper triangulation algorithms should use *M* for computation instead of *G*(*M*) aided with theoretical results about *G*(*M*). Constructing tree representations in order to minimally triangulate a graph without explicitly computing the fill edges was studied in [19] in order to achieve a faster time bound, and it would be interesting to see if these ideas can be extended to find a faster construction algorithm for 3-state perfect phylogeny.

## Competing interests

The authors declare that they have no competing interests.

## Authors’ contributions

The authors wrote the paper. All authors read and approved the final manuscript.
